# Opportunities and Constraints in Characterizing Landscape Distribution of an Invasive Grass from Very High Resolution Multi-Spectral Imagery

**DOI:** 10.3389/fpls.2017.00890

**Published:** 2017-05-30

**Authors:** Iryna Dronova, Erica N. Spotswood, Katharine N. Suding

**Affiliations:** ^1^Department of Landscape Architecture and Environmental Planning, University of California, BerkeleyBerkeley, CA, United States; ^2^San Francisco Estuary Institute-Aquatic Science Center, RichmondCA, United States; ^3^Department of Ecology and Evolutionary Biology, University of Colorado Boulder, BoulderCO, United States

**Keywords:** medusahead, invasive species, remote sensing, very high resolution, OBIA, rangeland

## Abstract

Understanding spatial distributions of invasive plant species at early infestation stages is critical for assessing the dynamics and underlying factors of invasions. Recent progress in very high resolution remote sensing is facilitating this task by providing high spatial detail over whole-site extents that are prohibitive to comprehensive ground surveys. This study assessed the opportunities and constraints to characterize landscape distribution of the invasive grass medusahead (*Elymus caput-medusae*) in a ∼36.8 ha grassland in California, United States from 0.15m-resolution visible/near-infrared aerial imagery at the stage of late spring phenological contrast with dominant grasses. We compared several object-based unsupervised, single-run supervised and hierarchical approaches to classify medusahead using spectral, textural, and contextual variables. Fuzzy accuracy assessment indicated that 44–100% of test medusahead samples were matched by its classified extents from different methods, while 63–83% of test samples classified as medusahead had this class as an acceptable candidate. Main sources of error included spectral similarity between medusahead and other green species and mixing of medusahead with other vegetation at variable densities. Adding texture attributes to spectral variables increased the accuracy of most classification methods, corroborating the informative value of local patterns under limited spectral data. The highest accuracy across different metrics was shown by the supervised single-run support vector machine with seven vegetation classes and Bayesian algorithms with three vegetation classes; however, their medusahead allocations showed some “spillover” effects due to misclassifications with other green vegetation. This issue was addressed by more complex hierarchical approaches, though their final accuracy did not exceed the best single-run methods. However, the comparison of classified medusahead extents with field segments of its patches overlapping with survey transects indicated that most methods tended to miss and/or over-estimate the length of the smallest patches and under-estimate the largest ones due to classification errors. Overall, the study outcomes support the potential of cost-effective, very high-resolution sensing for the site-scale detection of infestation hotspots that can be customized to plant phenological schedules. However, more accurate medusahead patch delineation in mixed-cover grasslands would benefit from testing hyperspectral data and using our study’s framework to inform and constrain the candidate vegetation classes in heterogeneous locations.

## Introduction

Understanding spatial distribution of invasive plant species at the early stages of infestation is critical for exposing the drivers of their expansion and informing preventive management (Westbrooks, 2004; Mangla et al., 2011). The process of invasion is often non-linear in space and time (Coutts et al., 2011), and rapid transitions from smaller, scattered patches to larger monodominant areas pose significant challenges to their control (With, 2002; Regan et al., 2006). Such dynamics may depend on complex cross-scale ecological interactions and threshold behavior which ultimately affect broader-scale landscape composition, yet may be difficult to assess in their entirety (With, 2002; Mayer and Rietkerk, 2004; Peters et al., 2007; Suding and Hobbs, 2009). To uncover these changes and underlying thresholds, patch distribution needs to be characterized comprehensively at the “mesoscale” level of individual sites, ideally capturing most or all of the potential participants in the infestation. However, this task requires high level of spatial detail and at landscape extents that may be too large (>1 ha) and prohibitive for traditional field sampling.

This challenge has been increasingly addressed by the applications of remote sensing data to detect plant invaders at the scales from individual sites to broader regions (Pengra et al., 2007; Andrew and Ustin, 2008; Hestir et al., 2008; Laba et al., 2010; Bradley, 2014). However, common satellite and aerial image data with spatial resolution of ≥1–4 m are often too coarse to discern individual patches and accurately characterize infestations (Hulme, 2003). Recent advances in very high (<1 m) resolution (VHR) airborne piloted and unmanned vehicle sensing offer a promise to overcome these limitations and to facilitate site-level analyses of invasions with more customizable and spatially informative data (Laliberte and Rango, 2009; Rango et al., 2009; Anderson and Gaston, 2013; Wan et al., 2014). However, practical value of these data for the patch-scale analyses and monitoring are not yet well understood for a broad range of invaders, particularly those morphologically similar to local species. Furthermore, several important challenges may affect the success of these efforts.

One key issue is that the advantages of high spatial resolution often come at the cost of excessive and less relevant spatial detail, such as color variation caused by shadows and canopy gaps. This variation poses a major problem for pixel-based landscape classifications, leading to losses of accuracy and the infamous “salt-and-pepper” speckle (Blaschke and Strobl, 2001; Blaschke, 2010). Previous studies have addressed this issue by using object-based image analysis (OBIA) where the images are first segmented into smaller regions (objects) via some of many available segmentation techniques, and these objects are subsequently classified into cover types (Baatz and Schäpe, 2000; Clinton et al., 2010). In addition to spectral values, object shape, heterogeneity (texture) and spatial contextual relationships may aid in class discrimination. Texture, from simple object-level variance to more complex gray-level co-occurrence matrix (GLCM) measures, has been particularly useful to represent class-specific intrinsic spatial patterns in applications of high-resolution imagery (Laliberte and Rango, 2009; Kim et al., 2011), including plant invasion studies (e.g., Ge et al., 2006; Tsai and Chou, 2006; Boers and Zedler, 2008; Laba et al., 2010). However, the overall OBIA process may be significantly complicated by spatial heterogeneity of vegetation, leading to complex and difficult to generalize approaches to yield higher accuracy (Laba et al., 2010; Blanchard et al., 2011; Rokitnicki-Wojcik et al., 2011; Allard et al., 2012). Their advantages to rigorous automated methods such as novel machine-learning algorithms (Heumann, 2011; Dronova et al., 2012; Zhang and Xie, 2012) are not yet well understood and call for more comparative studies.

A second challenge is that spectral sensitivity of many present-day VHR sensors is often limited to broad bands of visible and near-infrared electromagnetic regions (Rango et al., 2009; Anderson and Gaston, 2013; Wan et al., 2014; Toth and Jozkow, 2016). In contrast, many successful analyses of invasive species have relied on narrowband hyperspectral data (Underwood et al., 2003; Pengra et al., 2007; Andrew and Ustin, 2008; Hestir et al., 2008; Santos et al., 2011), and hyperspectral capabilities have been generally superior to hyperspatial ones in such studies (Nagendra and Rocchini, 2008; Rocchini et al., 2015). However, the latter evidence was based on the shortcomings of pixel-based methods such as local spectral variability (Nagendra and Rocchini, 2008), which can be addressed by OBIA (Baatz and Schäpe, 2000; Blaschke and Strobl, 2001; Burnett and Blaschke, 2003). Furthermore, limitations associated with cost and availability of high-resolution hyperspectral platforms are still challenging for management with constrained budgets. Thus, the potential of spectrally limited hyperspatial images to reveal early-stage invasions using novel processing methods remains to be tested. This question bears high practical importance as cheaper, easily customizable options of low-altitude and unmanned aircraft systems (UAS) imagery are gaining popularity (Laliberte and Rango, 2009; Anderson and Gaston, 2013; Colomina and Molina, 2014).

In response to these unknowns, our study assessed the opportunities and constraints to characterize the site-scale distribution of the invasive medusahead (*Elymus caput-medusae*) in a California, United States grassland using very high resolution (0.15 m) aerial imagery. Although a number of exotic grasses are currently prevalent in California, medusahead is a particularly problematic invasive species because it is associated with losses in forage production, wildlife habitat and biodiversity (Nafus and Davies, 2014). This annual grass promotes its dominance via several physiological adaptations and accumulation of dense thatch (George, 1992; Davies, 2008; Davies and Svejcar, 2008; Cherr, 2009; Nafus and Davies, 2014). Its dispersal may be facilitated by epizoochory due to presence of cattle, though specific implications for spread and patch distribution are not yet known (Chuong et al., 2016). Yet, livestock do not consume medusahead once it has reached the flowering stage, and as a result, its invasion is associated with reduced rangeland productivity and economic losses to ranchers in California (Nafus and Davies, 2014). Furthermore, medusahead is currently expanding its range across the western United States, having infested >1,000,000 ha and spreading at a rate of ∼12% a year (Duncan et al., 2004), and is therefore an invasive species of critical management concern.

Importantly, medusahead exhibits distinct phenology by staying green later in the season than many other annual grasses, which offers a potential to detect it remotely even with broader-band visible and near-infrared data (Cherr, 2009; Ndzeidze, 2011). Previous remote sensing analyses of this species applied pixel-based methods to both VHR (0.42 m) aerial imagery (Ndzeidze, 2011) and coarser 30 m Landsat satellite data (Cherr, 2009). However, these studies analyzed broad spatial extents (>20,000 ha) and did not explicitly discuss individual patch mapping or detection challenges due to presence of phenologically similar vegetation, which indicates the need for more in-depth site-level analyses of this species. Thus, our specific objectives were: (1) To test the potential of VHR broadband visible/near-infrared (VNIR) remote sensing imagery to characterize patch distribution of medusahead at its late spring phenology; (2) To assess and compare several OBIA classification strategies including unsupervised, supervised and knowledge-based methods; and (3) To assess the relative benefits of including object texture for medusahead classification. We also aimed to identify the main challenges to high-resolution medusahead monitoring to better understand the long-term prospects of this methodology for grassland management and the key future research needs.

## Materials and Methods

### Study Area and Vegetation Field Surveys

**FIGURE 1:**
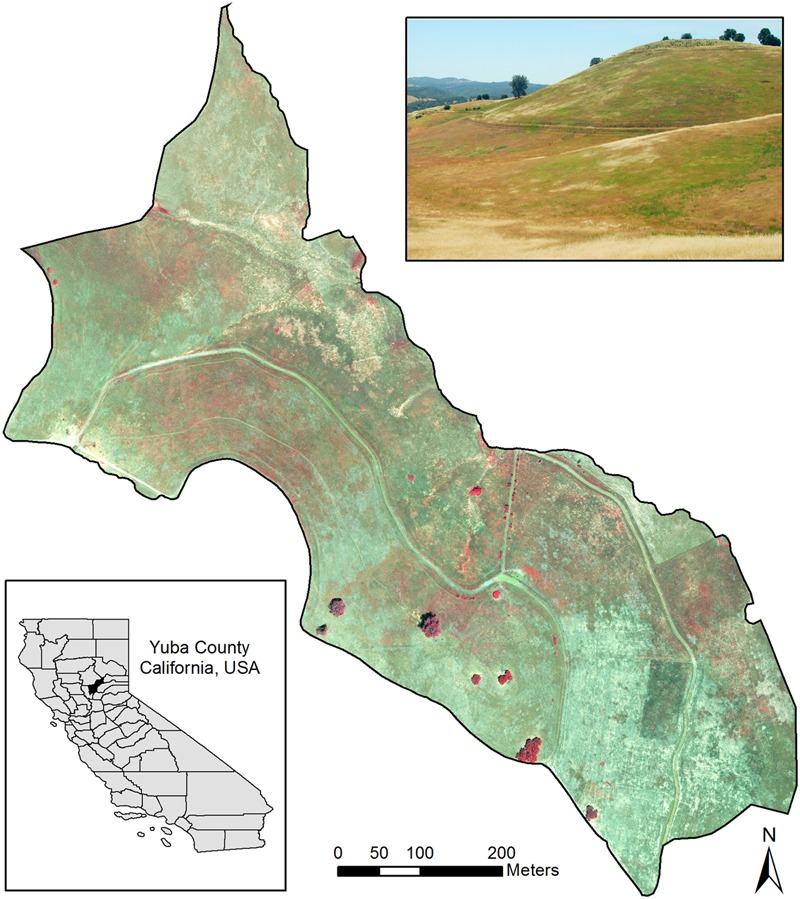
Study area at the Sierra Foothills Research Extension Center, Browns Valley, CA, United States. The main image shows a near-infrared, red and green band composite for the classified portion of the site. The photo inset shows an example of infested areas as seen on the ground, with medusahead and other green vegetation patches amidst senescent grasses.

This study focused on a ∼36.8 ha grassland (“Campbell”) site (**Figure 1**) at the University of California’s Sierra Foothills Research Experimental Center (SFREC) in Yuba County, California, United States (39°15.3′N, 121°17.1′W). This area has Mediterranean climate with dry hot summers and moist, cool winters. Grassland vegetation includes primarily naturalized exotic grasses, including slender wild oat (*Avena barbata*), brome (*Bromus* spp.), Italian ryegrass (*Lolium perenne*), bulbous canarygrass (*Phalaris aquatica*) and forbs such as clover (*Trifolium* spp.), vetch (*Vicia villosa*) and storksbill (*Erodium* sp.). Invasive medusahead and barbed goatgrass (*Aegilops triuncialis*) were present in several parts of the area, sometimes mixed with each other.

Several types of data on vegetation composition were collected in the field between mid-May and early June 2013. A comprehensive survey was performed using 20 transects placed as five groups of three, one group of four transects and one more separate transect in areas with different degree of infestation (or lack of thereof). The starting point and orientation for each group were chosen randomly. At each transect, ten 50 cm × 50 cm plots were surveyed at 10 m distance intervals to record the ID and relative percent cover of up to 10 most dominant vegetation species. After the surveys, plot locations were georeferenced using the Trimble GeoXH geographic positioning system (GPS) with the post-differential correction accuracy of ≤0.3 m.

Whenever the transects crossed medusahead patches in the field, start and end points of these intersections were recorded as the distance from a preceding plot to obtain a sample of field patch dimensions. In this assessment, medusahead “patches” were considered to be separate if the distance between them exceeded 20 cm. Later these intersections were converted from to a digital line vector dataset in ArcGIS v.10 (Esri Inc.) software using the plot GPS locations. Additional visual surveys were performed on several occasions during the study by walking around the site and taking photographs and GPS records of the off-transect vegetation examples for supplementary reference information.

Using these field data, we designated 300 location samples based on both transect data and additional surveys as reference and training data for the subsequent classifications. These samples were chosen to represent the instances of high percent cover of medusahead and other vegetation as closely as possible. The set included 50 samples of medusahead, Italian rye grass, vetch and clover-brome each, 40 samples of wild oat and 30 samples of less common barbed goatgrass and canarygrass each.

### Remote Sensing Data and Preliminary Assessment of Vegetation Distribution

Very high resolution (0.15 m) aerial imagery was acquired for May 19, 2013 at the stage of a pronounced phenological contrast between green medusahead and senescent dominant annual grasses. These data were collected by Eagle Digital Imaging Inc. using Canon 5D Mark 2 cameras and delivered as an orthorectified, mosaicked and radiometrically normalized product with three visible [red (600–680 nm), green (520–580 nm), blue (450–520 nm)] and one near-infrared (720–900 nm) electromagnetic bands. Prior to designing the object-based classification, field data were spatially overlaid with this image to visually examine the patterns of species co-occurrence and potential challenges to mapping.

**FIGURE 2:**
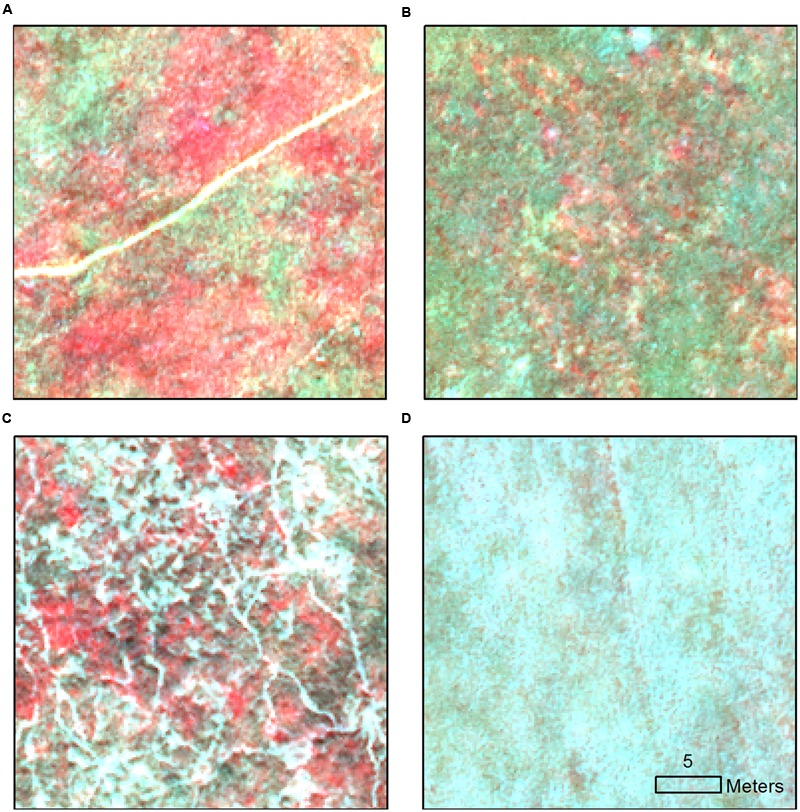
Examples of characteristic local spectral patterns shown in the near-infrared image composite for **(A)** medusahead and clover-brome; **(B)** canarygrass within senescent grasses; **(C)** vetch within clover-brome and Italian rye grass matrix and **(D)** medusahead within taller senescent wild oat.

This assessment revealed that several non-target vegetation types were also still green at the time of the study. Their spectral values at the pixel level were similar to green medusahead; however, some of them displayed distinct local patterns (**Figure 2**). Specifically, canarygrass occurred in the northwestern part of the study area as disaggregated green clumps within other senescent grasses. The mixtures of clover, brome and Italian rye grass formed areas with intermediate levels of greenness similar to lower-density medusahead, and often mixed with the latter. However, when the density of Italian ryegrass was high, these communities exhibited visually unique patterns with high local heterogeneity (**Figure 2**). Green barbed goatgrass was present at the site as either monodominant patches or mixed with local grasses and sometimes medusahead. Finally, vetch (**Figure 2**) was concentrated as very bright green patches primarily in the clove-brome areas and did not co-occur with medusahead in our sample data. These patterns suggested that textural metrics should be included in the subsequent image classifications in addition to spectral variables.

### General Classification Strategy and Image Segmentation

Given the potential confusion among medusahead and co-occurring green vegetation types, we compared three object-based image classification strategies: unsupervised, simple “single-run” supervised and more complex hierarchical object-based classifications described below. Unsupervised classification did not require training samples and allowed testing the possibility to detect medusahead with statistically evaluated clusters in the multi-dimensional space of discriminating input variables. Supervised single-run classification was used to test whether medusahead could be instantaneously discriminated from other vegetation types based on the class training samples. Finally, the hierarchical approach was developed to incorporate the local context of medusahead distribution into the process of its detection and discrimination from other vegetation.

Each classification was preceded by the segmentation of the image into objects to be used as mapping units. The input image layers for this step included four original image bands and two derived layers: a normalized difference vegetation index (NDVI; the normalized difference of near-infrared and red bands) to highlight vegetation greenness, and a local Moran’s I coefficient (Anselin, 1995) estimated using a 3 × 3 moving window as a metric of local heterogeneity. For unsupervised and single-run supervised classifications, primitive objects were generated using multi-resolution segmentation (MRS; Baatz and Schäpe, 2000) in eCognition software v.8.8 (Trimble Inc.). This procedure used low weights for shape and compactness of 0.1 each to emphasize spectral variables in object generation and the scale parameter value of 10 determined as the scale of substantial decline in the local spectral variance using the Estimation of Scale Parameter (ESP) tool (Dragut et al., 2010, 2014). For the hierarchical classification, we also used ESP to determine several nested scales of potential importance. Scale 390 corresponding to broad landscape units with different levels of heterogeneity and scale 176 capturing dominant vegetation communities were selected as the starting point for the hierarchical analysis (see Hierarchical Classification below).

In these approaches, a variety of object-level attributes were used to facilitate class discrimination. In supervised and hierarchical methods, spectral and textural attributes were selected based on the minimal overlap of class training samples within eCognition’s Sample Editor tool. Spectral variables included object-level means of the individual input image bands, while textural variables included standard deviations of the input image bands and more advanced GLCM measures (Haralick et al., 1973). Pilot analysis revealed that standard deviations of blue and green bands and all-direction GLCM Entropy of near-infrared and green bands were the most useful at both coarser and finer object scales. For the consistency, the unsupervised approaches used the same spectral and texture metrics. Finally, in the hierarchical classification class identities at higher levels of the object hierarchy were used to guide and constrain class assignments at the lower levels. Prior to all classifications, objects corresponding to trees and bright wetland vegetation near the stream beds were separately delineated and excluded from the analysis.

### Unsupervised Classification

Unsupervised object-based classification was implemented using a simple *k*-means clustering approach in Weka software (Frank et al., 2016). Two sets of object attributes were compared: spectral variables alone and together with texture metrics. To evaluate the suitable number of clusters, multiple runs of the algorithm were performed from 10 to 30 clusters, and within-object variance was computed for each run as an indicator of cluster tightness. While overall this metric declined with greater number of clusters, it occasionally exhibited local peaks that were interpreted as potentially meaningful changes in landscape structure. Two such peaks corresponded to the sets of 12 and 18 clusters that were selected for medusahead classification with spectral and spectral-texture attributes. Clusters likely to contain medusahead were identified both visually and by the spatial overlap with training sample locations.

### Single-Run Supervised Classification

Supervised object-based classifications were implemented in eCognition v.8.8 software by allocating primitive objects into candidate classes in a single run using a one-at-a-time set of discriminating attributes. To date, there has not been a clear consensus on which supervised algorithms are consistently the best for mapping vegetation in mixed-cover landscapes (Dronova et al., 2012; Zhang and Xie, 2012). Therefore, we compared three different machine-learning algorithms [*k*-nearest neighbor (KNN), Bayesian and support vector machine (SVM) with linear kernel] with two sets of discriminating features (spectral-only and spectral with texture as in the unsupervised clustering). Each of these was run using one simple and one more complex set of vegetation classes including, respectively, three (medusahead, other green, other non-green) or seven (medusahead, clover-brome, Italian ryegrass-brome, canarygrass, vetch, barbed goatgrass and wild oat) categories. All these methods used the same set of primitive training objects containing the locations of training samples for their specific classes.

### Hierarchical Classification

**FIGURE 3:**
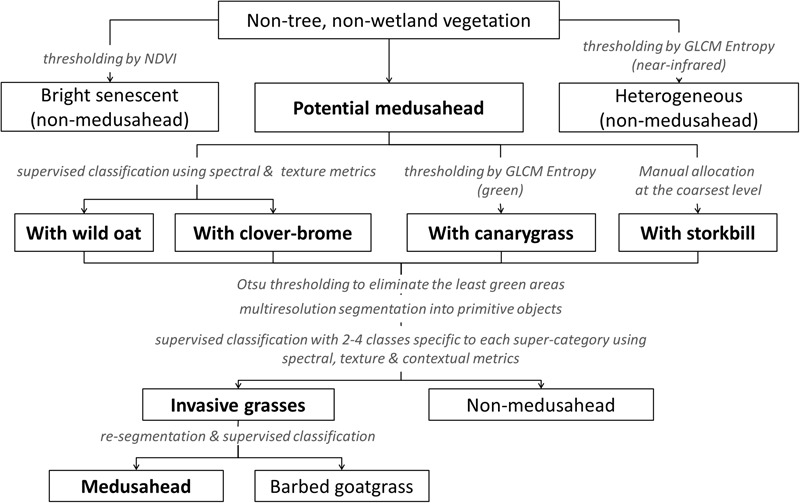
Major classes and steps in the knowledge-based hierarchical classification of medusahead.

A supervised/knowledge-based hierarchical classification was also tested as an alternative to single-run approaches when different classes may require different sets of discriminating features. Our hierarchical strategy combined automatic supervised classification with knowledge-based decision rules (**Figure 3**) that were informed by a series of pilot analyses using mathematical thresholding, trial-and-error and sample comparison in eCognition’s Sample Editor. Our final results included three algorithms with the same general procedure (**Figure 3**) but different supervised classifiers: KNN, SVM, and Bayesian, similar to single-run procedures.

At the coarsest level (determined by MRS at scale 390), we first isolated objects that were least likely to contain medusahead due to low NDVI and distant location from the known infested portions of the study area. Next, we isolated heterogeneous objects dominated by vetch, clover-brome and Italian rye grass mixtures based on their unique texture best represented by GLCM Entropy of the near-infrared band. In the field observations, these vetch associations did not include medusahead, consistent with prior uses of vetch as a medusahead control species (McLauchl et al., 1970).

The remaining objects, potentially containing medusahead, were classified at the next segmentation level (determined by MRS at scale 176) into broad classes representing associations with other dominant species (**Figure 3**). Areas with canarygrass were too similar spectrally to medusahead mixtures with clover-brome and could only be distinguished based on the GLCM Entropy of the green band. A small portion of the site contained green storksbill in the subcanopy of senescent grasses, which could not be easily separated with a spectral or textural threshold; these areas were labeled manually at the coarsest segmentation level. The remaining objects were then classified into medusahead associations with wild oat and clover-brome using object means of the red, green, blue and near-infrared bands and standard deviations of blue and green bands.

Next, these broader medusahead associations were classified into “invasive grasses” (medusahead and barbed goatgrass together) and other “non-medusahead.” To account for potentially non-uniform presence and greenness of medusahead among these groups, objects representing broader associations were split into three categories of greenness by thresholding the NDVI band using automatic Otsu method (Otsu, 1979) in Matlab software (version 2015a, MathWorks Inc.). The least green category in each group was isolated as non-medusahead (**Figure 3**). The remaining greener objects were reclassified into invasive grasses and other classes depending on their composition, and at the final step invasive grasses were further classified into medusahead and barbed goatgrass using object means for red and NDVI bands and GLCM Entropy of the near-infrared band.

### Classification Accuracy Assessment

Classification accuracy and uncertainty were evaluated for different classification outputs in two major ways focusing on medusahead as the target class. First, we performed a fuzzy accuracy assessment (Gopal and Woodcock, 1994) which was preferable to “hard” approaches (Congalton and Green, 2009) due to heterogeneity and common mixing of vegetation types. This analysis used a set of test samples designated from the field survey plots and field-visited locations not used in classification training. Most of these had mixed composition with more than one species as potential mapping candidate; thus, based on the field-recorded percent cover, each test plot was assigned one vegetation type as the “best” choice and, if applicable, another 1 or 2 classes as “acceptable.” When medusahead was present as a subdominant species with ≥5% cover, it was recorded as an “acceptable” candidate to test the possibility of its detection at lower densities. Overall, this test set included 150 samples, 50 of which had medusahead as the best candidate, 50 were non-medusahead and the other 50 were non-medusahead with medusahead as “acceptable.” Given potential geographic positioning errors, their locations were converted to 1 m × 1 m squares centered on the surveyed points and spatially overlaid with each classification outcome in ArcGIS v.10 software (Esri Inc.).

The following accuracy metrics were then computed for medusahead class following traditional and fuzzy approaches by adapting the methods in Gopal and Woodcock (1994):

(1) The proportion of test samples classified as medusahead having medusahead as the best reference class (“MAX” metric);
(2) The proportion of test samples classified as medusahead having medusahead as the acceptable reference class (“RIGHT” metric);
(3) The difference between (2) and (1) as the indicator of accuracy improvement by considering the fuzzy landscape composition (MAX-RIGHT);
(4) The proportion of test sample squares with medusahead as the best class which included classified medusahead within the square (“Producer’s accuracy” metric).

It is important to note that this set of metrics provides similar information about medusahead classification accuracy as conventional accuracy matrices (Congalton and Green, 2009), but it also reveals additional information about the error sources (Gopal and Woodcock, 1994). Specifically, the MAX metric is identical to user’s accuracy which is related to the error of commission and “overclassification” of a given class at the expense of others, while Producer’s accuracy metric describes the representation of reference samples and is related to the error of omission (Congalton and Green, 2009). The RIGHT metric proposed for heterogeneous landscapes shows to what extent classification results represent the target class when it is present but not necessarily dominant or exclusive (Gopal and Woodcock, 1994), while the MAX-RIGHT difference further shows to what extent user’s accuracy improves when such heterogeneity is taken into account. Hence, the combined use of these metrics is expected to provide both “hard” estimates of accuracy and error and the information about potential effects of distributional or spectral heterogeneities on medusahead discrimination.

**FIGURE 4:**
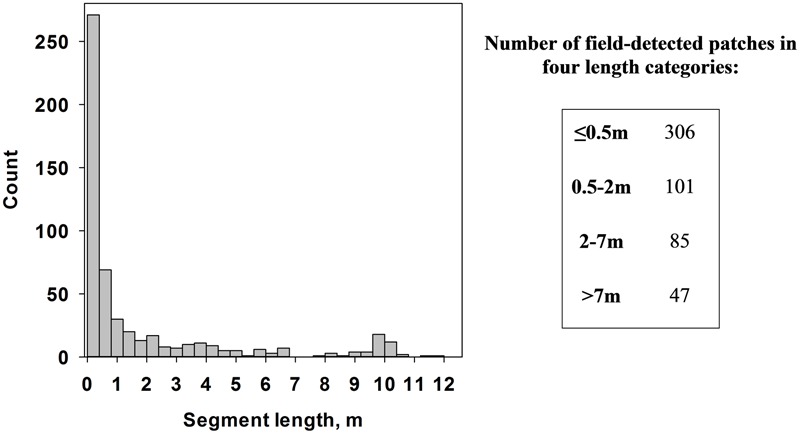
Distribution of medusahead patch segment lengths in the field transects (left) and counts of medusahead segments in four major length categories (right).

Second, medusahead patches produced by different algorithms were overlaid with field transect segments (**Figure 4**) in ArcGIS software to assess their spatial match. Because classification uncertainty could be related to patch size, we defined four categories of segment length from the statistical distribution of length in the field dataset (≤0.5 m, 0.5–2 m, 2–7 m and >7 m) and quantified two sets of metrics for each of them. The first set represented counts and average lengths of segments produced by the intersection of classified medusahead extents and field segments, to assess the potential under-prediction of the latter. The second set included counts and average lengths of segments produced by the intersection of classified medusahead patches and full transect lines, to assess the potential over-prediction of the field segments. Because transect locations and field-determined medusahead sections could be affected by positioning errors and the surveyors’ judgment of patch identities, results of this assessment were interpreted with caution, focusing on the relative differences among classification outcomes.

## Results

### Classification Outcomes and Fuzzy Accuracy of Different Methods

Medusahead classification accuracy differed among the compared approaches (**Table 1**), but a number of similar strengths and limitations were evident in their results. The MAX metric describing the match between classification result and medusahead as a primary class (**Table 1**) rarely exceeded 50%. However, when medusahead was considered as an acceptable, even if not the best, candidate class, these accuracies improved, reaching 63–83% (**Table 1**). Producer’s accuracy ranged from 56 to 90% in supervised methods and was even higher in the unsupervised approaches (**Table 1**). Including texture variables increased producer’s accuracy in most cases (**Table 1**), highlighting the importance of texture in reducing the error of medusahead omission at the high spatial resolution of the aerial data. However, the effects of texture on the error of commission were not uniform among methods (**Table 1**), showing improvement mainly for SVM and unsupervised 18-cluster approaches and the opposite or no effect in other methods.

**Table 1:** Fuzzy accuracy metrics for medusahead classification by different methods (MAX and RIGHT metrics denote the proportions of test samples classified as medusahead having medusahead as the best and acceptable class, respectively; Producer’s accuracy metric indicates the proportion of test samples with medusahead as the best class which included classified medusahead).

Method	With texture	MAX	RIGHT	MAX-RIGHT	Producer’s accuracy
Unsupervised, 12 clusters	-	0.36	0.68	0.32	0.96
Unsupervised, 12 clusters	Yes	0.34	0.70	0.36	1.00
Unsupervised, 18 clusters	-	0.35	0.68	0.33	0.98
Unsupervised, 18 clusters	Yes	0.35	0.71	0.36	1.00
Supervised KNN, 3 classes	-	0.45	0.81	0.36	0.66
Supervised KNN, 3 classes	Yes	0.44	0.77	0.33	0.64
Supervised KNN, 7 classes	-	0.45	0.78	0.33	0.66
Supervised KNN, 7 classes	Yes	0.45	0.77	0.33	0.68
Supervised SVM, 3 classes	-	0.23	0.67	0.44	0.44
Supervised SVM, 3 classes	Yes	0.40	0.66	0.26	0.56
Supervised SVM, 7 classes	-	0.35	0.66	0.31	0.64
Supervised SVM, 7 classes	Yes	0.45	0.73	0.28	0.90
Supervised Bayesian, 3 classes	-	0.44	0.73	0.29	0.82
Supervised Bayesian, 3 classes	Yes	0.44	0.71	0.27	0.88
Supervised Bayesian, 7 classes	-	0.52	0.83	0.31	0.60
Supervised Bayesian, 7 classes	Yes	0.48	0.76	0.32	0.76
Hierarchical with KNN	Yes	0.40	0.69	0.29	0.76
Hierarchical with SVM	Yes	0.33	0.63	0.30	0.58
Hierarchical with Bayesian	Yes	0.46	0.76	0.30	0.74

The substantial differences of 0.26–0.44 between MAX and RIGHT metrics (**Table 1**) indicate that many of the medusahead’s overpredictions (assignments to objects with other classes as the best candidates), were still a match for its presence and thus informative for the purpose of detection. However, some of the test samples dominated by medusahead were consistently misclassified, especially those with green barbed goatgrass and clove-brome mixtures. These outcomes represented classification error resulting from the similarity of these classes. Consistent with this observation, the accuracy metrics substantially improved when the accuracies and errors of medusahead and barbed goatgrass were combined for 7-class supervised and hierarchical approaches (**Table 2**). It was also challenging to detect medusahead in the understory of taller senescent grasses such as wild oat because such locations often did not appear green enough. Occasional non-uniformities in medusahead phenology also contributed to classification errors, with several patches observed in the field being partially or completely senescent and thus confused with other grasses.

**Table 2:** Combined accuracies for medusahead and barbed goatgrass in supervised seven-class and hierarchical classifications.

Method	With texture	MAX	RIGHT	Producer’s
Supervised KNN, 7 classes	-	0.51	0.83	0.96
Supervised KNN, 7 classes	Yes	0.52	0.82	0.86
Supervised SVM, 7 classes	-	0.49	0.77	0.87
Supervised SVM, 7 classes	Yes	0.54	0.85	0.92
Supervised Bayesian, 7 classes	-	0.62	0.90	0.73
Supervised Bayesian, 7 classes	Yes	0.57	0.86	0.88
Hierarchical with KNN	Yes	0.51	0.80	0.78
Hierarchical with SVM	Yes	0.53	0.85	0.78
Hierarchical with Bayesian	Yes	0.53	0.85	0.84

### Differences in Classification Results among Specific Approaches

Among specific methods, unsupervised approaches produced the highest producer’s accuracy for medusahead test samples. However, low values of the MAX metric show that this was achieved at the expense of extreme overprediction: in all four algorithms only ∼1/3 of the reference samples classified as medusahead were actually dominated by it (**Table 1**). With both spectral and spectral-texture attribute sets, clusters capturing medusahead locations automatically included areas dominated by vetch and other green species. No cluster in any of the runs was unique to medusahead or other green classes alone, which significantly limits the value of unsupervised classification outcomes for patch distribution analysis.

**FIGURE 5:**
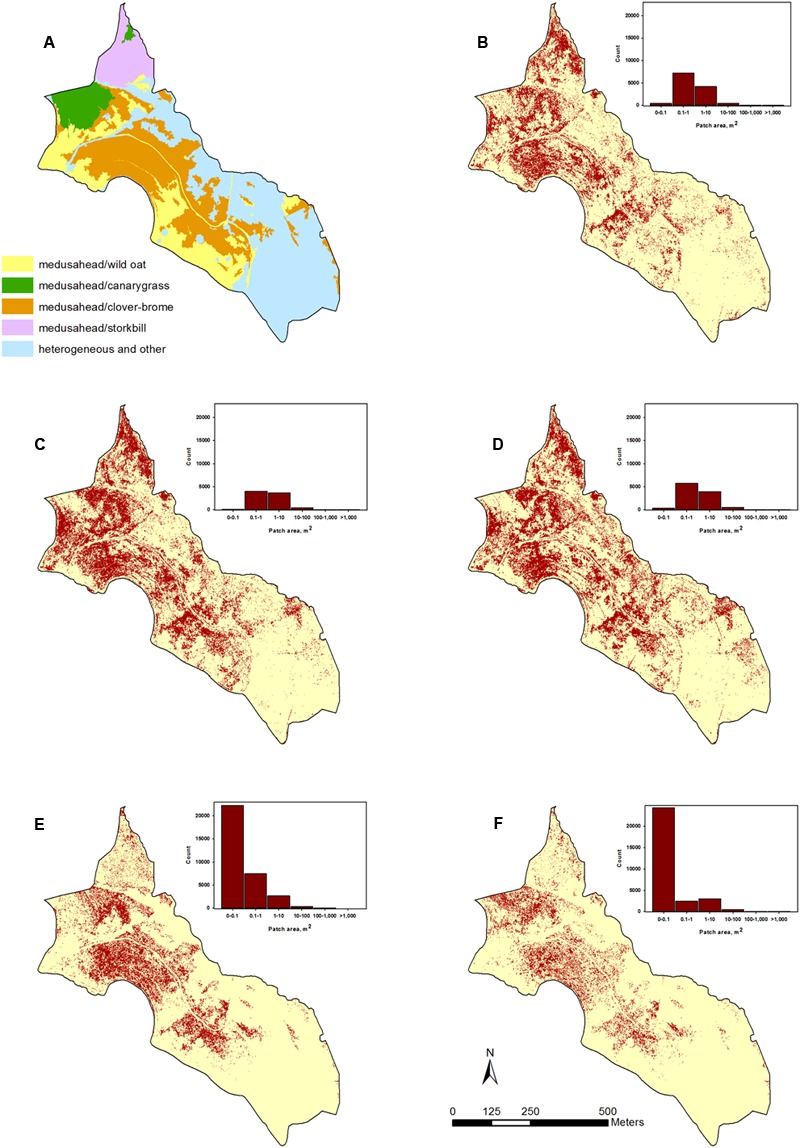
Examples of classified medusahead using: **(A)** coarser level of the hierarchical classification showing potential medusahead associations with other dominant species; **(B)** supervised SVM algorithm using seven vegetation classes with spectral and texture attributes; **(C,D)** supervised Bayesian algorithm using three vegetation classes with and without texture attributes, respectively; and **(E,F)** finer level of the hierarchical classification using KNN and Bayesian supervised classifiers, respectively.

In contrast, supervised single-run and hierarchical classification outcomes had a lower proportion of test medusahead samples classified as such based on producer’s accuracy (**Table 1**), but often a higher proportion of classified medusahead samples matching test data as the best or acceptable class (MAX and RIGHT metrics in **Table 1**). Spatial distribution of medusahead among the methods also varied (**Figure 5**), and their relative performance strongly depended on the choice of class categories and object attributes. The most balanced results with relatively high values of both metric types (**Table 1**) were from the SVM algorithm using seven classes with texture (**Figure 5B**) and supervised Bayesian using three classes both with texture (**Figure 5C**) and without (**Figure 5D**). Using three instead of seven classes reduced the MAX and producer’s accuracy metrics (i.e., increased the errors of both omission and commission) in KNN and SVM approaches (**Table 1**), indicating that a simpler classification scheme was not necessarily beneficial for medusahead detection. However, for Bayesian algorithm producer’s accuracy was substantially higher in the three-class scheme, while MAX and RIGHT were higher in the seven-class scheme (**Table 1**). This result indicates that Bayesian method produced a better match to reference samples in a simpler class set, but a lower chance of overprediction in the seven-class scenario.

Finally, the hierarchical approaches differed in their results, with the highest accuracy produced with Bayesian in the supervised classification steps (**Table 1**), and the lowest – with SVM, which considerably misclassified medusahead as barbed goatgrass at the final step (**Figure 3**). Not surprisingly, SVM accuracy substantially improved when medusahead and barbed goatgrass accuracies were considered together (**Table 2**), and in these results, texture provided further improvement of both user’s and producer’s accuracies only in case of SVM. Notably, accuracy of the best hierarchical outcome did not surpass the best results from the single-run classifications, though exceeded most of them (**Table 1**). However, spatial distribution of the classified medusahead with hierarchical methods including KNN (**Figure 5E**) and Bayesian (**Figure 5F**) algorithms highlighted important advantages of this method, such as restriction of class assignments based on the broader vegetation types (**Figure 5A**) and isolation of vetch-dominated areas and senescent grasslands. For instance, in single-run outcomes, misclassifications between medusahead and vetch resulted in spillover medusahead allocations (**Figures 5B–D**) within heterogeneous and other areas that were absent from the hierarchical result (**Figures 5A,E,F**). Also, in contrast to single-run methods, hierarchical classification outcomes included a greater number of smaller-sized (<0.1 m^2^ in area) patches (**Figures 5E,F**), likely due to the use of spectral thresholds at intermediate steps.

### Match between Classification and Field Transect-Based Patch Dimensions

**FIGURE 6:**
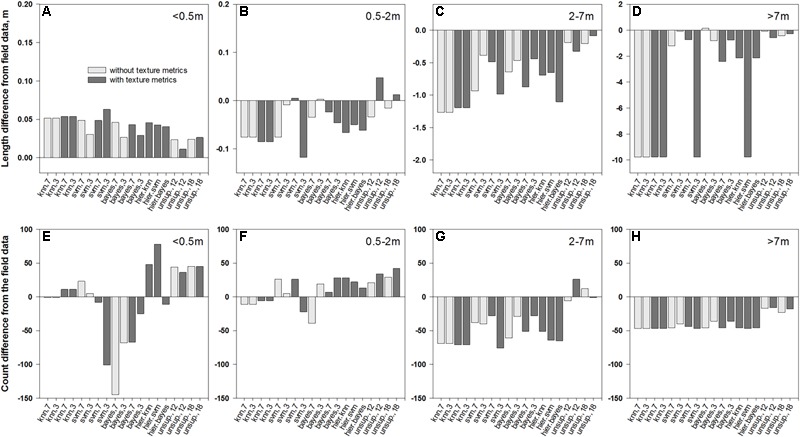
Differences in average length of patch segments **(A–D)** and their counts **(E–H)** between field data and their intersections with medusahead classification outcomes summarized for different patch sizes: **(A,E)**<0.5 m; **(B,F)** 0.5–2 m; **(C,G)** 2–7 m; and **(D,H)** >7 m.

**FIGURE 7:**
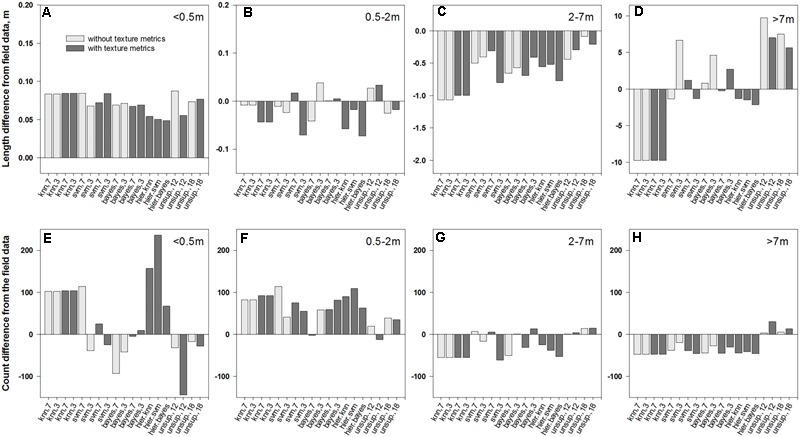
Differences in average length of patch segments **(A–D)** and their counts **(E–H)** between field data and the intersections of medusahead classification outcomes with full transect lines summarized for different patch sizes: **(A,E)**<0.5 m; **(B,F)** 0.5–2 m; **(C,G)** 2–7 m; and **(D,H)** >7 m.

The overlay of medusahead classifications with field-based transect segments (**Figure 4**) and full transect lines revealed that performance of different methods varied among the four length categories (**Figures 5–7**). The original field dataset had almost three times as many patches ≤0.5 m in length than in 0.5–2 m and 2–7 m categories, and even fewer larger patches >7 m (**Figure 4**). With all the methods, the average length of the smallest resulting segments (≤0.5 m) was higher than in the field alone (**Figures 6A, 7A**), while their counts increased or decreased depending on the method (**Figures 6E, 7E**). This result reflected classification difficulties to detect the smallest patches visible in the field, particularly those within senescent taller grasses. In contrast, average length and counts of the largest segments (2–7 m and >7 m) were considerably under-estimated compared to the field data (**Figures 6C,D,G,H**); and even missed by some methods such as KNN. This outcome was likely attributed to misclassification of some medusahead objects along the transect lines, which, in turn, could have resulted from the non-uniform cover and density within larger patches.

Overall, classification methods which produced the closest match to the field segments in a given category were not always the same that performed best in terms of fuzzy accuracy (**Table 1**). However, this result could have been affected by positioning errors, and in the statistics for the intersections with full transects the single-run SVM and Bayesian algorithms with seven classes and texture and the hierarchical approach using KNN were frequently among the closest (**Figure 7**). Notably, all four unsupervised classifications showed a close match in average length to the original patches (**Figures 6A–D**); however, this occurred due to their massive overestimation of medusahead extents, particularly for the largest patches (**Figures 7D,H**). The statistics for the intersection of medusahead segments with full transects also showed greater counts for the patches of 0.5–2 m size (**Figure 7H**), which could have been caused both by the under-estimation of larger patches and by the misclassifications of other vegetation types as medusahead.

## Discussion

### Strengths and Challenges in Characterizing Site-Scale Medusahead Distribution

The infestation and spread of noxious exotic weeds are affected by complex interactions among plant individuals, species, site factors and disturbance, which ultimately become manifested in the spatial distribution and dynamics of their patches (With, 2002; Hamada et al., 2007; Laca, 2009; Santos et al., 2011). Comprehensive site-scale analyses of patch distributions are thus important for addressing questions about cross-scale interactions and critical thresholds in the invasion process to inform ecosystem management (George, 1992; Peters et al., 2006; Suding and Hobbs, 2009). However, as such questions continue to emerge in various ecosystems, traditional field approaches become increasingly limited compared to new remote sensing opportunities for assessing these distributions at the whole-site scales and in high spatial detail (Kellner et al., 2011; Bradley, 2014; Asner et al., 2015; Rocchini et al., 2015). Yet, this potential comes at a cost of a higher landscape data complexity that needs to be addressed in order to make reliable inference about the distribution and ecological performance of invaders.

Our study at the California SFREC site provides the first, to our knowledge, effort to characterize the site-scale patch distribution of medusahead by applying OBIA to VHR visible and near-infrared aerial data. Overall, this strategy appears to be useful for detecting medusahead’s general spread and identifying the hotspot locations of the larger, established patches. Most classification methods assigned medusahead to >75% and up to 83% of test samples where it was an acceptable vegetation type (**Table 1**), which is comparable to previous applications for medusahead detection from VHR imagery in south central Oregon, United States, similarly relying on medusahead phenology as a basis for detection (Ndzeidze, 2011). These results are encouraging given the challenges posed by medusahead’s resemblance to several resident species, in contrast to studies where unique morphological and spectral characteristics of invaders make them easier to be identified exclusively (Lass et al., 1996; Hunt et al., 2004; Hamada et al., 2007; Hestir et al., 2008).

At the same time, heterogeneity in patch size and local percent cover of medusahead had important implications for the classification uncertainty. Small patches were not likely to include enough pixels to display class-specific “characteristic” texture, and thus spectral similarity between medusahead and other vegetation was a major challenge in their representation. Under limited dispersal, metrics of distance to larger, more easily identifiable patches could have been used to address this issue. However, such metrics were not as useful in this study site due to grazing-facilitated dispersal (Chuong et al., 2016). Thus, small patches were more likely to be missed or overestimated with most methods, while their validation could be especially sensitive to geolocation errors.

Dispersal also likely contributed to local mixing of medusahead with other green and senescent vegetation which could have affected the under-estimation of larger, less uniform patches. This outcome raises a pertinent question of how patches should be defined as ecologically relevant units in such a mixed-vegetation setting (Peters et al., 2006). Medusahead boundaries were particularly uncertain when its density was low over relatively large extents, as in clover-brome communities or in the understory of taller senescent grasses. Because even 0.15 m spatial resolution was not sufficient to detect individual plants in these mixtures, any contiguous area with low densities could hypothetically represent a single “patch,” potentially overestimating the extent of medusahead alone and reducing the agreement between field and remotely sensed patch sizes.

Such an overestimation should be interpreted with caution, however, because from the management perspective it is often desirable to assess the infestation as fully as possible. Thus, some error in the form of “false positive” detections may be considered less problematic than “false negatives” missing true invader occurrences, although it still has its cost (Hulme, 2003). However, overprediction on a massive scale, such as by the unsupervised classifications in our study, can make the results virtually useless for patch-level inference, especially when paralleled by misclassification of the reference medusahead samples. These results also indicate that phenological contrast may not be always a reliable basis for medusahead detection and delineation in mixed-species grasslands (Ndzeidze, 2011), and other green species need to be explicitly considered in these efforts.

### Practical Lessons from the Image Analysis Methods

Our results highlight important benefits and shortcomings of the tested OBIA methodology for very high resolution medusahead detection. The key advantage of this framework was in the possibility to incorporate non-spectral attributes, particularly texture, in class discrimination. The potential utility of texture was evident in the improvement of classification results with some of the methods and manifested itself in two major ways. First, the small pixel size relative to the target landscape entities allowed to capture some of their unique intrinsic spectral variability and thus to facilitate classification under limited spectral data, similar to other OBIA applications in complex vegetated landscapes (Dubuisson-Jolly and Gupta, 2000; Laliberte and Rango, 2009; Laba et al., 2010). Second, texture was also useful at the coarser levels of the hierarchical classification which included smaller patches of green cover types together with their “matrix” of senescent grasses. For canarygrass and vetch, this strategy provided the only feasible way to isolate them from other green vegetation types and thus to reduce the chance of false detection outside of their primary extents.

The importance of GLCM Entropy on top of simple standard deviation metrics further indicates that more structured local spatial patterns provide useful indicators of vegetation types in addition to their intrinsic variability *per se* (Laliberte and Rango, 2009; Allard et al., 2012). However, even these advanced metrics could not fully resolve the challenge of class confusion, particularly for medusahead and barbed goatgrass that were similar in both phenology and local pattern. Furthermore, the intrusions of these invaders into “characteristic” local patterns of other species, such as canarygrass, constrained the potential of coarser-level texture metrics to inform the identity of finer-level green patches.

The choice of a specific classification technique may also strongly affect medusahead detection and representation of its patch structure. Under the absence of other green vegetation, medusahead could have been delineated using simple thresholding, such as Otsu method (Otsu, 1979) applied to bands or indices representing variation in greenness (Torres-Sanchez et al., 2015). However, in this landscape such methods would inevitably group multiple green types together, thus overestimating the target species’ extent. Similarly, the unsupervised *k*-means clustering could not effectively discriminate among medusahead and phenologically similar vegetation types, even with the inclusion of texture. As a result, greener clusters overpredicted medusahead patches by encompassing other vegetation types. This outcome is important because unsupervised techniques may appear tempting when the field data are limited yet high spatial resolution allows recognizing dominant vegetation types based on their characteristic patterns. However, discerning medusahead patches within those types may be difficult with VNIR data alone, even by an expert. Thus from a practical standpoint, unsupervised techniques may not be sufficient for detecting individual patches, and high match of their outcomes to reference medusahead locations may be misleading.

In contrast, supervised methods were more effective at differentiating medusahead from other vegetation by learning from training data. Novel machine-learning algorithms, such as three methods used in our study, have been increasingly applied for vegetation analyses due to their potential to outperform traditional methods (e.g., Dronova et al., 2012; Zhang and Xie, 2012, 2013). However, our results indicate that relative success of these methods varied depending on a specific classification strategy. Furthermore, all of them were constrained by medusahead’s phenological similarity to other green species and high intrinsic heterogeneity of vegetation types, leading to incorrect detections of medusahead outside of its primary range, based on the test sample accuracy. Simultaneous improvement of MAX, RIGHT and producer’s accuracy metrics in the combined results for medusahead and barbed goatgrass (**Table 2**) highlights the mutual confusion of these grasses as a particularly critical source of error in medusahead detection with VNIR imagery. This issue was especially relevant to SVM classifier which relies on the marginal contrasts among class sample distributions and often failed to differentiate among these two species despite the tendency to overclassify medusahead in this study.

In turn, the hierarchical approaches provided a useful capacity to navigate the landscape complexity and to inform local vegetation classification by its coarser-scale spectral and textural “context.” Hierarchical approaches have often been praised for the possibility to enhance classification accuracy by compartmentalizing both the landscape structure and the classification process (Krause et al., 2004; Wagner, 2009; Kamal and Phinn, 2011). In our study, these benefits reduced the misclassification spillover effects, though at the cost of missing potential infestations in excluded areas. However, comparable accuracy to single-run methods raises a question to what extent the algorithm complexity (**Figure 3**) and greater cost of time for its development were justified by the outcomes. There may be endless possibilities for classification refinement and expansion of such stepwise procedures, which may ultimately reduce their reproducibility (Blanchard et al., 2011; Rokitnicki-Wojcik et al., 2011; Allard et al., 2012). Thus, to be useful and practical for medusahead detection and monitoring, hierarchical approaches need to be relatively constrained and should not extensively rely on user-defined, image-specific thresholds that may vary with data properties.

### Implications for the Future Work on Medusahead Detection

Limitations encountered in this analysis provide important insights into the general task of mapping invasive grasses and future research needs for medusahead and similar species. The key challenge related to medusahead’s similarity to barbed goatgrass and several other species calls for exploring alternative types of remote sensing information to enhance their discrimination in the future. For example, some efforts have successfully used multi-angular spectral reflectance to detect medusahead based on its unique changes in leaf orientation during the reproductive period (Naupari et al., 2013). However, variable density of medusahead in our study area and its relatively common presence in the subcanopy of taller grasses would likely reduce the utility of structural information at this and similar sites.

Alternatively, richer multi-spectral and hyperspectral information should be tested for detecting medusahead with electromagnetic regions sensitive to its unique characteristics. With sufficiently high spatial resolution, such analyses may help to uncover smaller patches or presence of medusahead in mixed communities. Although instruments with such capabilities are still not common, the emerging technology can make them more available in the near future (Lucieer et al., 2014; Toth and Jozkow, 2016). However, the diversity and heterogeneity of grassland vegetation would remain an important challenge for hyperspectral analyses due to a large and spatially varying pool of candidate species as spectral classes. Resolving this issue may require measures to restrict the candidate sets across the landscape, such as with multiple-endmember spectral mixture analysis at coarser resolutions (MESMA; Okin et al., 2001). This task could be facilitated by cost-effective object-based classifications of multispectral data into regions representing potential medusahead associations with other species, such as in our study. Thus combining our framework with hyperspectral analyses would be a useful strategy to enhance the inference of medusahead’s distribution and its spatial associations with other vegetation types. This direction should be explored to better understand the mesoscale drivers of the early-stage infestations and to enhance the cost-effective monitoring strategies in grasslands.

## Conclusion

Our results indicate that applying OBIA framework to very high resolution VNIR imagery is a useful strategy for detecting general extents and hotspots of invasive medusahead infestations in the mixed-cover grasslands. An important prerequisite for such mapping is the time of high phenological contrast between green medusahead and other dominant grasses. Among several compared unsupervised, single-run supervised and hierarchical classification approaches, the accuracy of medusahead detection varied and generally increased when simple and advanced texture metrics were used together with spectral variables. The worst performance was shown by the unsupervised *k*-means clustering algorithms that were not able to disentangle medusahead and other green species as separate clusters and thus overestimated the areas with potential medusahead presence. More customized and complex hierarchical classification procedures were not able to exceed the accuracy of the single-run methods; however, they somewhat reduced the spillover misclassifications of medusahead outside of its primary extents. Reducing vegetation classes to general green and senescent vegetation categories with single-run approaches resulted in lower accuracy compared to more specific classes, suggesting the need to account for vegetation complexity in order to facilitate class discrimination. The key limitations in this analysis included spectral confusions of medusahead with other green vegetation types, particularly invasive barbed goatgrass, and variable degree of mixing with other species. These issues likely hampered the ability of most classification methods to accurately reproduce medusahead patch structure, particularly for the smallest patches that tended to be missed or overestimated in size, and the largest patches that were fragmented and/or underestimated. Collectively, results suggest that the key benefits of the VNIR very high spatial resolution data include the detection of local infestation hotspots and capturing medusahead associations with other vegetation types based on their unique textural patterns. These outcomes, in turn, offer promise to guide and effectively constrain more specific species-level detection from hyperspectral platforms that should be tested in future work.

## Author Contributions

ID, ES, and KS conceived of and planned out this study. ES and ID performed the field data collection. ID performed the analysis of remote sensing data and wrote the manuscript. ES and KS contributed to interpretation of the results and manuscript editing.

## Conflict of Interest Statement

The authors declare that the research was conducted in the absence of any commercial or financial relationships that could be construed as a potential conflict of interest.
